# Supplementary material for: Misoprostol regulates Bnip3 repression and alternative splicing to control cellular calcium homeostasis during hypoxic stress
Source: Cell Death Discov. 2018 Sep 21;4:98. doi: 10.1038/s41420-018-0104-z (PMC6155004; doi:10.1038/s41420-018-0104-z)
Supplement: Supplementary file 1 — Supplemental Data [file 41420_2018_104_MOESM1_ESM.pdf]

Supplemental

Human BNIP3 FL (NM_004052):

```
  1 - ATGTCGCAGAACGGAGCGCCCGGGATGCAGGAGGAGAGCCTGCAGGGCTCCTGGGTAGAA - 60
  1 - M  S  Q  N  G  A  P  G  M  Q  E  E  S  L  Q  G  S  W  V  E   - 20

 61 - CTGCACTTCAGCAATAATGGGAACGGGGGCAGCGTTCCAGCCTCGGTTTCTATTTATAAT - 120
 21 - L  H  F  S  N  N  G  N  G  G  S  V  P  A  S  V  S  I  Y  N   - 40

121 - GGAGACATGGAAAAAATACTGCTGGACGCACAGCATGAGTCTGGACGGAGTAGCTCCAAG - 180
 41 - G  D  M  E  K  I  L  L  D  A  Q  H  E  S  G  R  S  S  S  K   - 60

181 - AGCTCTCACTGTGACAGCCCACCTCGCTCGCAGACACCACAAGATACCAACAGAGCTTCT - 240
 61 - S  S  H  C  D  S  P  P  R  S  Q  T  P  Q  D  T  N  R  A  S   - 80

241 - GAAACAGATACCCATAGCATTGGAGAGAAAAACAGCTCACAGTCTGAGGAAGATGATATT - 300
 81 - E  T  D  T  H  S  I  G  E  K  N  S  S  Q  S  E  E  D  D  I   - 100

301 - GAAAGAAGGAAAGAAGTTGAAAGCATCTTGAAGAAAAACTCAGATTGGATATGGGATTGG - 360
101 - E  R  R  K  E  V  E  S  I  L  K  K  N  S  D  W  I  W  D  W   - 120

361 - TCAAGTCGGCCGGAAAATATTCCCCCCAAGGAGTTCCTCTTTAAACACCCGAAGCGCACG - 420
121 - S  S  R  P  E  N  I  P  P  K  E  F  L  F  K  H  P  K  R  T   - 140

421 - GCCACCCTCAGCATGAGGAACACGAGCGTCATGAAGAAAGGGGGCATATTCTCTGCAGAA - 480
141 - A  T  L  S  M  R  N  T  S  V  M  K  K  G  G  I  F  S  A  E   - 160

481 - TTTCTGAAAGTTTTCCTTCCATCTCTGCTGCTCTCTCATTTGCTGGCCATCGGATTGGGG - 540
161 - F  L  K  V  F  L  P  S  L  L  L  S  H  L  L  A  I  G  L  G   - 180

541 - ATCTATATTGGAAGGCGTCTGACAACCTCCACCAGCACCTTTTGA - 585
181 - I  Y  I  G  R  R  L  T  T  S  T  S  T  F  *   - 200
```

Human BNIP3ΔExon2 (MF593120):

```
  1 - ATGTCGCAGAACGGAGCGCCCGGGATGCAGGAGGAGAGCCTGCAGGCCCACCTCGCTCGC - 60
  1 - M  S  Q  N  G  A  P  G  M  Q  E  E  S  L  Q  A  H  L  A  R   - 20

 61 - AGACACCACAAGATACCAACAGAGCTTCTGAAACAGATACCCATAGCATTGGAGAGAAAA - 120
 21 - R  H  H  K  I  P  T  E  L  L  K  Q  I  P  I  A  L  E  R  K   - 40

121 - ACAGCTCACAGTCTGAGGAAGATGATATTGAAAGAAGGAAAGAAGTTGAAAGCATCTTGA - 180
 41 - T  A  H  S  L  R  K  M  I  L  K  E  G  K  K  L  K  A  S  *   - 60
```

Human BNIP3Δ(Exon2+Exon3)

```
  1 - ATGTCGCAGAACGGAGCGCCCGGGATGCAGGAGGAGAGCCTGCAGGTCTGAGGAAGATGA - 60
  1 - M  S  Q  N  G  A  P  G  M  Q  E  E  S  L  Q  V  *  G  R  *   - 20

 61 - TATTGAAAGAAGGAAAGAAGTTGAAAGCATCTTGA - 95
 21 - Y  *  K  K  E  R  S  *  K  H  L *   - 40
```

Mouse Bnip3 FL (NM_009760) :

```
  1 - ATGTCGCAGAGCGGGGAGGAGAACCTGCAGGGCTCCTGGGTAGAACTGCACTTCAGCAAT - 60
  1 - M   S   Q   S   G   E   E   N   L   Q   G   S   W   V   E   L   H   F   S   N   - 20

 61 - GGCAATGGGAGCAGCGTTCCAGCCTCCGTCTCTATTTATAATGGTGACATGGAAAAAATA - 120
 21 - G   N   G   S   S   V   P   A   S   V   S   I   Y   N   G   D   M   E   K   I   - 40

121 - CTGTTGGATGCCCAGCATGAATCTGGACGAAGTAGCTCCAAGAGTTCTCACTGTGACAGC - 180
 41 - L   L   D   A   Q   H   E   S   G   R   S   S   S   K   S   S   H   C   D   S   - 60

181 - CCACCTCGCTCCCAGACACCACAAGATACCAACAGAGCTGAAATAGACAGCCACAGCTTT - 240
 61 - P   P   R   S   Q   T   P   Q   D   T   N   R   A   E   I   D   S   H   S   F   - 80

241 - GGCGAGAAAAACAGCACTCTGTCTGAGGAAGATTATATTGAGAGAAGAAGAGAAGTTGAA - 300
 81 - G   E   K   N   S   T   L   S   E   E   D   Y   I   E   R   R   R   E   V   E   - 100

301 - AGTATCCTGAAGAAAAACTCAGATTGGATATGGGATTGGTCAAGTCGACCAGAAAATATT - 360
101 - S   I   L   K   K   N   S   D   W   I   W   D   W   S   S   R   P   E   N   I   - 120

361 - CCCCCCAAGGAGTTCCTTTTTAAACACCCGAAGCGCACAGCTACTCTCAGCATGAGAAAC - 420
121 - P   P   K   E   F   L   F   K   H   P   K   R   T   A   T   L   S   M   R   N   - 140

421 - ACAAGCGTTATGAAGAAAGGGGGAATTTTCTCAGCAGACTTTCTGAAGGTTTTCCTTCCA - 480
141 - T   S   V   M   K   K   G   G   I   F   S   A   D   F   L   K   V   F   L   P   - 160

481 - TCTCTGTTACTGTCTCATCTGCTGGCCATTGGCTTGGGGATCTACATTGGAAGGCGTCTG - 540
161 - S   L   L   L   S   H   L   L   A   I   G   L   G   I   Y   I   G   R   R   L   - 180

541 - ACAACTTCCACTAGCACCTTCTGA - 564
181 - T   T   S   T   S   T   F   *   - 200
```

Mouse Bnip3ΔExon3 (MF156210) :

```
  1 - ATGTCGCAGAGCGGGGAGGAGAACCTGCAGGGCTCCTGGGTAGAACTGCACTTCAGCAAT  - 60
  1 - M   S   Q   S   G   E   E   N   L   Q   G   S   W   V   E   L   H   F   S   N   - 20

 61 - GGCAATGGGAGCAGCGTTCCAGCCTCCGTCTCTATTTATAATGGTGACATGGAAAAAATA  - 120
 21 - G   N   G   S   S   V   P   A   S   V   S   I   Y   N   G   D   M   E   K   I   - 40

121 - CTGTTGGATGCCCAGCATGAATCTGGACGAAGTAGCTCCAAGAGTTCTCACTGTGACAGT  - 180
 41 - L   L   D   A   Q   H   E   S   G   R   S   S   S   K   S   S   H   C   D   S   - 60

181 - CTGAGGAAGATTATATTGAGAGAAGAAGAGAAGTTGAAAGTATCCTGA   - 228
 61 - L   R   K   I   I   L   R   E   E   E   K   L   K   V   S   *   - 80
```

Mouse Bnip3Δ(Exon2+Exon3)

```
  1 - ATGTCGCAGAGCGGGGAGGAGAACCTGCAGGTCTGAGGAAGATTATATTGAGAGAAGAAG - 60
  1 - M   S   Q   S   G   E   E   N   L   Q   V   *   G   R   L   Y   *   E   K   K   - 20

 61 - AGAAGTTGAAAGTATCCTGA-
 21 - R   S   *   K   Y   P  *
```
